# Antidiabetic effect of bio-enhanced preparation of turmeric in streptozotocin-nicotinamide induced type 2 diabetic Wistar rats

**DOI:** 10.1016/j.jaim.2021.04.010

**Published:** 2021-08-02

**Authors:** Vinay Kumar Sayeli, Ashok K. Shenoy

**Affiliations:** a Department of Pharmacology, Apollo Medical College, Chittoor, Andhra Pradesh, 517127, India; b Department of Pharmacology, Kasturba Medical College, Mangalore, Manipal Academy of Higher Education, Manipal, Karnataka, 575001, India

**Keywords:** Diabetes, Turmeric, Curcumin, Pancreas distribution, Insulin resistance, Beta cell activity

## Abstract

**Background:**

Poor oral bioavailability of curcumin, the active ingredient in turmeric, has limited its therapeutic use in various diseases including diabetes mellitus (DM).

**Objective(s):**

The present study was aimed at evaluating and comparing the antidiabetic activity as well as pharmacokinetic profile of two turmeric extracts.

**Materials and methods:**

Rats were divided into seven groups (n = 6) including Normal control (NC), Diabetic control (DC), two standard control groups- Glibenclamide (GLIB) 5 mg/kg and Metformin (MET) 500 mg/kg, two bio-enhanced turmeric extract (BTE) treated groups (BTE-30 (30 mg/kg), BTE-60 (60 mg/kg)) and one regular turmeric extract treated (RTE) group RTE-30 (30 mg/kg). Treatment was given orally for 30 days. Streptozotocin (60 mg/kg) and Nicotinamide (110 mg/kg) were administered intraperitoneally to induce diabetes. Fasting blood glucose (FBG), oral glucose tolerance test at 60 min and 120 min (OG1 and OG2) were analysed at baseline and at the end of study on Day 29. FBG, fasting serum insulin, and concentration of curcumin and its derivatives present in pancreas were analysed at the end of study on Day 30.

**Results:**

Turmeric extract treated groups showed significant (p < 0.05) blood glucose lowering effect, when compared with DC group. FBG, OG1 and OG2 readings were found significantly (p < 0.05) higher in RTE-30 treated group when compared with BTE-30 treated groups. Turmeric extracts showed improved beta-cell function, insulin sensitivity and decreased insulin resistance. BTE-30 had more pancreatic bioavailability of curcumin than RTE-30.

**Conclusion:**

Turmeric extracts demonstrated an antidiabetic effect in streptozotocin-nicotinamide induced type 2 diabetic Wistar rats. BTE extract was found to be an effective agent as compared to RTE in controlling hyperglycemia.

## 1 Introduction

Diabetes mellitus (DM) is the most common chronic metabolic disease affecting almost 6% of the world population [1]. It is one of the critical non-communicable diseases which is a significant threat to global health [2]. Prevalence of DM is expected to rise up to 4.4% by the year 2030 from 2.8% in the year 2000 [3]. It is associated with a state of chronic hyperglycaemia which can cause long term injury to specific tissues, notably the retina, kidney, nerves, and arteries [2].

Maintaining blood glucose in normal range and preventing complications due to long-term hyperglycaemia are the important goals of therapy in management of DM [4]. The common therapeutic approach of DM is to control blood glucose with lifelong use of antidiabetic drugs. Though, there are various classes of approved oral antidiabetic drugs, most of them cause unwanted side-effects, drug interactions and are expensive [5]. Hence, many of the institutes and pharmaceutical industries are involved in drug development to find the molecules with good therapeutic value and decreased adverse events [4]. Effective herbal medicines account for minimal adverse effects and decrease the cost of therapy. Hence, despite of considerable progress in the treatment of DM, with expensive synthetic drugs, there is always a quest for herbal remedies.

Natural products and their derivatives have received considerable attention for the management of DM. One of the most studied natural product and popular spice in Asian cuisine is turmeric. It is derived from the root of the plant *Curcuma longa* L., which has been mentioned in the treatment of diabetes in traditional Ayurvedic and Chinese medicine for thousands of years [6]. Curcumin is the main curcuminoid found in turmeric. It is widely consumed and generally believed to be beneficial for human health. Curcumin extract from rhizomes of turmeric has been shown to possess anti-inflammatory, anticancer and antidiabetic properties as well [7].

However, turmeric formulations have low bioavailability, and this limits their therapeutic use. Several formulations were developed to increase the bioavailability of curcumin. Bio-enhanced preparation of turmeric in which curcumin is reconstituted with essential oil of turmeric has been found to have better bioavailability, i.e., up to 6.93 fold than normal curcumin [8]. However, there is no evidence demonstrating the antidiabetic effect of bio-enhanced preparation of turmeric with increased bioavailability.

Broadly two types of experimental models are available for inducing DM in rodents. Models of first type include induction of diabetes in normal rodents (either by decreasing the number of beta cells by surgery/by administration of pancreatic toxins like Alloxan, Streptozotocin (STZ) or by administration of substances like Glucocorticoids, high Fructose diet which can induce insulin resistance) and the models of second type include animals that are genetically predisposed to development of DM like Bio Breeding (BB) rat (mechanism in this model is development of auto-immune destruction of pancreatic β cells) and Cohen rat (mechanism in this model is development of insulin resistance after high sucrose diet administration) [9]. Genetically predisposed experimental animal models are expensive and not widely available [10,11].

STZ and Alloxan are pancreatic toxins that can be used to develop type 1 and type 2 diabetes in rodent models [12]. STZ was found to be a better substance than alloxan to induce diabetes [10].

STZ when administered to rats at a dose of 50 mg/kg, i.p., can produce type 1 diabetes while lowering STZ to 40 mg/kg does not produce effective diabetes mellitus [13]. In Streptozotocin-Nicotinamide (STZ- NA) model, STZ induces diabetes as it has cytotoxic action on pancreatic beta-cells, whereas NA is given prior to administration of STZ to these rats which partially protect these cells against STZ. Hence, administration of NA prevents complete damage of β-cells and this model can act as type 2 diabetic model [12]. Wistar rats used in this study were induced with diabetes by using the STZ- NA model [[14], [15], [16]]. STZ, 60 mg/kg and NA 110 mg/kg are the doses used in this study to induce type 2 experimental diabetes.

The objective of the study was to evaluate the antidiabetic activity of BTE in diabetic Wistar rats and to compare its efficacy with (RTE) and standard drugs (Glibenclamide (GLIB) and Metformin (MET)). The effect of test drugs on serum insulin levels were evaluated. The presence of curcumin and its derivatives in pancreatic tissue of the animals were also analysed.

## 2 Materials and methods

### 2.1 Study Materials

#### 2.1.1 Turmeric extracts

RTE and BTE used in the study were extracted from botanically identified rhizomes of turmeric (*C*. *longa* L., Zingiberaceae family). 1.0 kg of dried turmeric rhizome was extracted with ethyl acetate to form turmeric oleoresin from which curcuminoid crystals were precipitated under low temperature, dried and powdered to obtain 30 g of turmeric extract (RTE) standardised with 95% curcuminoids. BTE is a blend of turmeric extract with 95% curcuminoids not less than 86% and turmeric essential oil made by steam distillation standardised with 45% aromatic turmerone not less than 7%. Turmeric extracts were provided by Arjuna Natural Pvt. Ltd., India as gift samples. High performance liquid chromatography (HPLC) with PDA detector is the most common method for the determination of curcuminoids in turmeric extracts. Due to the very labile characteristics of curcuminoids, C18 columns were preferred for HPLC analysis. Due to certain advantages over traditional HPLC, Ultra Performance Liquid Chromatography (UPLC) method for analysis of curcuminoids in turmeric extract was used. 25 mg of turmeric extract was made up with acetone in a 25 mL volumetric flask; from that, 1 mL was again pipetted out and diluted using acetone to 5 mL volumetric flask and filtered through a 0.22 μm filter before injection. C18 column of dimension 2.6 μm (100∗4.6 mm) was used with 40% THF and 60% water (containing 1% citric acid, pH adjusted to 3.0 using concentrated potassium hydroxide solution) as mobile phase, with 0.5 mL/min flow rate, column temperature 40 °C, injection volume 2 μL and detection was done at 420 nm wavelength. In C18 columns, depending on their polarity or hydrophobicity, curcuminoids were separated. Based on differentials partitioning between a mobile phase and a stationary phase, curcumin elutes first at 6.80–6.90 min, Demethoxycurcumin (DMC) elutes at 7.35–7.45 min while Bisdemethoxycurcumin (BDMC) elutes last at 7.85–7.95 min. For analysis of aromatic turmerone, C18 column of dimension 2.6 μm (100∗4.6 mm) with 85% acetonitrile in water as mobile phase with flow rate of 0.2 mL/min, injection volume of 2 μL at 25 °C and detection was done at 254 nm wavelength. The percentage of curcuminoids and aromatic turmerone was calculated against reference standards.

#### 2.1.2 Standard drugs

GLIB (Cadila Health Care Pvt. Ltd. Batch no: GCLIMA04A) and MET (Glyciphage 500 mg tablets, Franco India Pharmaceuticals Pvt. Ltd. Batch no: GA15012) were the standard drugs used.

#### 2.1.3 Chemicals used for induction of DM

STZ (Hi media, Lot no: 0000204509), NA (Hi media, Lot no: 0000103591), Citric acid (Ranbaxy Fine Chemicals Ltd. Product no: c0340), Trisodium citrate (Nice chemicals, Batch no: 605025), Tween 80 (Hi media, Lot no: 0000172935), Sodium chloride (Nice chemicals, Batch no: 909333) and Anesthetic ether (Narsons pharma, Batch no: AE01).

#### 2.1.4 Reference standards used for estimating curcumin and its derivatives in pancreatic tissues of Wistar rats

Curcumin (98%, HWI pharma services GmbH, Germany), DMC and BDMC (95%, Sigma–Aldrich, USA), Curcumin Glucuronide (CG) (94.6%, TLC Pharmaceutical Standards Ltd, Canada) and Curcumin Sulphate (CS) (87.1%, TLC Pharmaceutical Standards Ltd, Canada). All chemicals used for the biochemical estimations were of analytical reagent grade.

### 2.2 Animals

A total of 42 male Wistar rats were used in this study and they were obtained from Central Animal House of our institution. Rats were kept in clean polypropylene cages at a temperature of 24–26 °C. Three rats were housed per each cage. Standard care was provided to the animals as per the Committee for the Purpose of Control and Supervision of Experiments on Animals (CPCSEA) guidelines. All animals were allowed to acclimatize for one week before the experiment. Experiments were conducted only during light phase of cycle (09:00–17:00 h). The study was initiated only after obtaining written approval from the Institutional Animal Ethics Committee of Kasturba Medical College, Mangalore (CPCSEA number of Institutional Animal Ethics Committee: 213/PO/Re/S/2000/CPCSEA).

### 2.3 Study procedure

#### 2.3.1 Diabetes induction procedure

This study was done at the Department of Pharmacology, Kasturba Medical College, Mangalore, Manipal Academy of Higher Education, Manipal, Karnataka. The diabetic model that we have used in this study was STZ-NA model [14]. DM was induced in male Wistar rats by giving NA and STZ injections, intraperitoneally.

A single dose of NA 110 mg/kg, dissolved in freshly prepared 0.9% normal saline, in a volume of 4.8 mL/kg body weight, was given intraperitoneally, Later, after 15 min STZ, 60 mg/kg, dissolved in freshly prepared 0.1 M Citrate buffer with a pH 4.5, in a volume of 4.8 mL/kg body weight, was administered intraperitoneally. Citrate buffer (0.1 M), which has been used as solvent for mixing STZ, was made by dissolving solution of trisodium citrate (294.10 mg in 100 mL of distilled water) with citric acid solution (210.10 mg in 100 mL of distilled water) to attain pH 4.5. Ice was kept around the glass beaker containing Citrate buffer to maintain citrate buffer at lower temperatures. Normal control rats were administered with Citrate buffer alone intraperitoneally.

On the 4^th^ day, after the diabetes induction procedure rats with fasting blood glucose value (FBG) > 126 mg/dl or Oral glucose tolerance test (OGTT) first hour or second hour reading (OG1/OG2)> 200 mg/dl were considered as diabetic rats and they were included in the study.

#### 2.3.2 Treatment schedule

Wistar rats were divided into seven groups, each group had six rats. Treatment for all the groups was started on the 4th day after diabetes induction procedure. The same was considered as Day 1 for treatment and continued for 30 days. The animals were treated for 30 days. The treatment schedule of various groups were as follows: Normal control (NC) (1 mL of 1% Tween 80, per oral), Diabetic control (DC) (1 mL of 1% Tween 80, per oral), two Standard control groups were treated with GLIB (5 mg/kg, per oral) and MET (500 mg/kg, per oral), two test groups were treated with bio-BTE (30 mg/kg, per oral and 60 mg/kg, per oral) and one test group was treated with RTE (30 mg/kg, per oral). The vehicle used for the study in all the groups were 1% Tween 80. Oral feeding of drugs was done using steel oral gavage tube (16 G, 7.6 cm). During the treatment period, rats also received standard pelleted chow (VRK Nutritional solutions) and water *ad libitum* during treatment.

#### 2.3.3 Estimation of fasting blood glucose, oral glucose tolerance test, serum insulin readings during study

FBG was estimated in all rats before administration of STZ and NA and had FBG level less than 126 mg/dl. OGTT was performed in all groups of rats on Day 1 (4^th^ day after induction) and also on Day 29 of treatment. For performing OGTT, rats were fasted overnight for 12 h were challenged with a glucose solution, a dose of 2 g/kg body weight, by oral intubation. FBG values were measured at 0 min (before glucose administration) and OGTT readings (OG1 and OG2) were measured at 60 and 120 min after administration of glucose solution. FBG, OG1 and OG2 values were estimated from blood drops that were obtained from rat tail tip using Accu-Check Glucometer. On Day 30 of treatment, FBG was measured and after anaesthetizing rats with ether, blood was collected from overnight fasted rats using cardiac puncture into red-coloured procoagulant vacutainers and centrifuged at 3000 rpm for 7 min to obtain serum. These serum samples were stored at −80 °C before insulin levels were assayed using DRG Rat insulin ELISA Kit. Homeostasis Model Assessment (HOMA) 2 calculator version 2.2.3 [17] was used to calculate Insulin resistance (HOMA –IR), beta-cell function (%B) and insulin sensitivity (%S) with FBG and fasting serum insulin obtained on Day 30.

#### 2.3.4 Body weight measurement

Body weight of the rats was measured before induction (on the day of induction), on Day 1 of treatment and also on Day 30 of treatment. Percentage change in body weight was calculated using the formula

$$\frac{\left(\text{Body}\;\text{weight}\;\text{on}\;\text{Day}\;30\;\text{of}\;\text{treatment}-\text{Body}\;\text{weight}\;\text{on}\;\text{Day}\;1\;\text{of}\;\text{treatment}\right)}{\left(\text{Body}\;\text{weight}\;\text{on}\;\text{Day}\;1\;\text{of}\;\text{treatment}\right)}\times 100$$

#### 2.3.5 Analysis of curcumin, derivatives and metabolites

Quantification of curcumin, DMC, BDMC in free form and its metabolites (CG and CS) in pancreas were performed via liquid chromatography with tandem mass spectrometry (MS/MS) (Shimadzu, Marlborough, MA, USA). Pancreas was thawed, homogenised in ammonium acetate buffer (1 M, pH 4.6, 10% wv1) and extracted with ethyl acetate, which had been pre-saturated with ice-cold propan-2-ol [17]. Urolithin B was used as internal standard and chromatographic separation was performed on a 50 mm × 2.1 mm i.d., 2.5 μm, XBridge BEH C18 column Waters, (Milford, MA, USA) with mobile phase A [acetonitrile containing 0.1% (v/v) formic acid] and mobile phase B [water containing 0.1% (v/v) formic acid]. Elution was completed using a 9-min isocratic program (43% A; 57% B) operating with a 0.2 mL/min flow rate with an 8 μL injection volume. The column temperature was maintained at 40 °C [18]. The retention times for curcumin glucuronide, curcumin sulphate, bisdemethoxycurcumin, demethoxycurcumin and curcumin were 1.18, 1.65, 2.82, 3.09, 3.41 min respectively. All calibration curves were linear using weighted linear least squares regression 1/X2. The calibration curves for curcumin, desmethoxycurcumin, bisdemethoxycurcumin, curcumin-O-glucuronide and curcumin-O-sulfate showed linearity over the concentration range of 1–500, 0.5–250, 0.05–25, 1–600 and 1–100 ng/mL respectively. Representative chromatograms are provided as Supplementary file 1 and 2.

#### 2.3.6 Statistical analysis

One-way ANOVA followed by post hoc analysis with Tukey test was conducted to evaluate statistically significant differences in the study variables between treatment groups. Pearson two-tailed correlation analysis was used to evaluate the correlation between serum insulin and IR; serum insulin and %B; and serum insulin & %S. SPSS version 21.0 was used to conduct statistical analysis in this study.

## 3 Results

At baseline or Day 1 of treatment (4 days after induction), blood glucose levels were found to be significantly (p < 0.05) elevated in diabetes-induced groups (DC, GLIB, MET, BTE-30, BTE-60, RTE-30) compared to NC group. All diabetes-induced rats met the inclusion criteria (FBG>126 mg/dl or OG1 reading >200 mg/dl). This observation concludes that the model used in this study was able to induce diabetes (state of hyperglycaemia) in all STZ-NA treated rats. At baseline, like FBG, OG1 and OG2 readings were also found to be elevated significantly in diabetes-induced groups (DC, GLIB, MET, BTE-30, BTE-60, RTE-30) when they were compared to the NC group (p < 0.05) (Table 1).

**Table 1:** Fasting Blood Glucose level, Oral Glucose Tolerance Test (first and second hour) (mg/dL).

Treatment groups	Day 1 Readings (baseline)			Day 29 Readings (after treatment)		
	FBG	OG1	OG2	FBG	OG1	OG2
	Mean ± SD	Mean ± SD	Mean ± SD	Mean ± SD	Mean ± SD	Mean ± SD
NC	85.17 ± 5.95^b^	98 ± 7.48^bcd^	88.33 ± 4.97^bcd^	84 ± 8.88^b^	97 ± 7.54^bc^	90 ± 6.99^b^
DC	125.33 ± 11.17^a^	395.17 ± 44.34^a^	338.00 ± 41.59^a^	396.33 ± 154.91^acd^	470.50 ± 131.09^acd^	418.67 ± 162.66^acd^
GLIB	130.5 ± 5.43^a^	420.67 ± 21.71^a^	358.50 ± 34.65^a^	95.17 ± 27.97^b^	154.17 ± 76.55^b^	117.50 ± 16.21^b^
MET	128.33 ± 5.32^a^	421.67 ± 39.52^a^	301.50 ± 64.05^a^	144.83 ± 53.86^b^	329.33 ± 12.91^abd^	203.67 ± 58.96^b^
BTE-30	132.67 ± 3.78^a^	390.67 ± 43.74^a^	325.17 ± 39.85^a^	81.17 ± 9.66^b^	240.33 ± 60.85^ab^	112.67 ± 6.31^b^
BTE-60	128.83 ± 7.22^a^	370.33 ± 64.76^a^	338.00 ± 41.59^a^	80.00 ± 3.29^b^	139.67 ± 41.31^b^	93.33 ± 12.13^b^
RTE-30	132.00 ± 6.99^a^	440.83 ± 32.73^a^	361.67 ± 86.86^a^	197 ± 19.42^bcd^	289.33 ± 83.61^abd^	281.67 ± 81.59^abcd^

^a^p < 0.05 compared to NC; ^b^p < 0.05 compared to DC; ^c^p < 0.05 compared to BTE-30; ^d^p < 0.05 compared to BTE-60.

NC = Normal Control; DC = Diabetic control; GLIB = Glibenclamide treated; MET = Metformin treated; BTE-30 and BTE-60 = Bioenhanced turmeric extract 30 mg/kg and 60 mg/kg treated; RTE-30 = Regular turmeric extract 30 mg/kg treated.

After treatment or on Day 29, significant (p < 0.05) increase in FBG value was observed in DC as compared to other treatment groups and NC group. FBG level observed in RTE-30 treatment group was found to be significantly greater than FBG levels observed in BTE-30 and BTE-60 treated groups (Table 1).

After treatment or on Day 29, OG1 and OG2 readings of DC group were also found to be significantly elevated when compared with other treatment groups (Table 1). OG1 reading of RTE-30 group was found to be significantly higher than the reading observed with BTE-60 group. OG2 reading of RTE-30 was found to be significantly higher than the readings observed with BTE-30 and BTE-60 treatment groups (Table 1).

Day 30 FBG level, fasting serum insulin levels and the parameters derived from these two variables using HOMA-2 calculator such as IR, %S and %B are summarized in Table 2. Serum insulin was found to be significantly elevated in GLIB group when compared with NC group among various treatment groups. Though the mean serum insulin readings in MET and RTE-30 treated groups is less than DC group, this observation was not statistically significant (Table 2).

**Table 2:** Day 30 Fasting Blood Glucose (FBG), Serum Insulin level, Insulin Resistance (IR), Insulin Sensitivity (IS), Beta cell function (%B), correlation of serum insulin with beta cell function, insulin sensitivity and insulin resistance among various treatment groups.

Day 30	FBG (mg/dL)	Serum Insulin (pmol/L)	Insulin Resistance (IR)	Insulin Sensitivity (%S)	Beta cell function (%B)	Serum Insulin vs %B	Serum Insulin vs %S	Serum Insulin vs IR
Treatment group	Mean ± SD	Mean ± SD	Mean ± SD	Mean ± SD	Mean ± SD	Correlation coefficient (p value)		
NC	83.50 ± 8.31^b^	71.26 ± 15.41	1.30 ± 0.30^b^	80.20 ± 17.69	138.10 ± 40.79^b^	0.74 (p = 0.09)	−0.98 (p < 0.001)	0.99 (p < 0.001)
DC	394.20 ± 18.64^acd^	52.87 ± 15.13^c^	3.75 ± 0.94^acd^	28.77 ± 10.48^d^	6.55 ± 2.00^acd^	0.98 (p < 0.001)	−0.50 (p = 0.31)	0.46 (p = 0.35)
GLIB	96.67 ± 26.49^b^	145.68 ± 41.06^abd^	2.70 ± 0.84^abd^	41.08 ± 16.03	183.13 ± 68.69^b^	−0.06 (p = 0.90)	−0.09 (0.002)	0.98 (p < 0.001)
MET	141.00 ± 52.33^abcd^	35.63 ± 16.81^c^	0.73 ± 0.31^bc^	160.70 ± 68.64^abcd^	42.03 ± 37.09^acd^	0.73 (p = 0.09)	−0.88 (p = 0.02)	0.98 (p < 0.001)
BTE-30	89.17 ± 9.00^b^	97.69 ± 38.30^b^	1.78 ± 0.67^b^	65.47 ± 31.47	149.40 ± 63.30^b^	0.84 (p = 0.04)	−0.96 (p = 0.003)	0.99 (p < 0.001)
BTE-60	80.50 ± 3.39^b^	58.30 ± 13.54	1.05 ± 0.26^b^	99.47 ± 25.17^b^	126.12 ± 24.48^b^	0.87 (p = 0.02)	−0.99 (p < 0.001)	0.99 (p < 0.001)
RTE-30	185.00 ± 12.43^abcd^	35.49 ± 6.64^c^	0.78 ± 0.14^b^	132.57 ± 30.39^bc^	17.65 ± 3.61^acd^	0.79 (p = 0.05)	−0.99 (p < 0.001)	0.98 (p < 0.001)

^a^p < 0.05 compared to NC; ^b^p < 0.05 compared to DC; ^c^p < 0.05 compared to BTE-30; ^d^p < 0.05 compared to BTE-60.

NC = Normal Control; DC = Diabetic control; GLIB = Glibenclamide treated; MET = Metformin treated; BTE-30 and BTE-60 = Bioenhanced turmeric extract 30 mg/kg and 60 mg/kg treated; RTE-30 = Regular turmeric extract 30 mg/kg treated.

IR was found to be significantly low in standard drug and test drug treated groups compared to DC group (Table 2). %S was found to be significantly elevated in MET, BTE-60 and RTE-30 treatment groups when compared with DC group (Table 2). Serum insulin has shown positive correlation with %B, negative correlation with %S and positive correlation of serum insulin is also seen with IR (Table 2).

The change in body weight is represented in Table 3. In our study, we observed that Day 30 body weight was decreased when compared with baseline among DC group, MET treated group and turmeric extracts treated groups (BTE-30, BTE-60 and RTE-30). Whereas, on Day 30 body weight was increased when compared with baseline in NC group and GLIB treated group (Table 3).

**Table 3:** Body weight of normal control, diabetic control and diabetic treated rats.

Treatment group	Day 1	Day 30	Percentage change in body weight on Day 30 when compared to baseline
	Mean ± SD	Mean ± SD	Gain (+)/Loss (−)
NC	181.83 ± 4.99	238.33 ± 17.49^bcd^	+31.07%
DC	184.50 ± 4.09	167.67 ± 5.39^a^	−9.12%
GLIB	179.67 ± 3.08	221 ± 8.07^abcd^	+23%
MET	183.67 ± 7.82	161.5 ± 7.74^acd^	−12.07%
BTE-30	182.67 ± 4.18	172.33 ± 4.23^a^	− 5.66%
BTE-60	185.17 ± 3.13	172 ± 6.20^a^	− 7.11%
RTE-30	177.83 ± 3.37	171.67 ± 4.18^a^	−3.46%

^a^p < 0.05 compared to NC; ^b^p < 0.05 compared to DC; ^c^p < 0.05 compared to BTE-30; ^d^p < 0.05 compared to BTE-60.

NC = Normal Control; DC = Diabetic control; GLIB = Glibenclamide treated; MET = Metformin treated; BTE-30 and BTE-60 = Bioenhanced turmeric extract 30 mg/kg and 60 mg/kg treated; RTE-30 = Regular turmeric extract 30 mg/kg treated.

Concentration of curcumin and its derivatives in pancreas at the end of the study on Day 30 in test drug treated groups (BTE-30, BTE-60 and RTE-30) groups are summarized in Table 4. When compared with RTE-30 treated group, BTE-30 and BTE-60 showed significant elevation of curcumin and its derivatives (Table 4).

**Table 4:** Concentration level of curcumin and its derivatives in pancreas.

Group	Curcumin (ng/gm)	DMC (ng/gm)	BDMC (ng/gm)	CG (ng/gm)	CS (ng/gm)
	Mean ± SE	Mean ± SE	Mean ± SE	Mean ± SE	Mean ± SE
BTE-30	160.50 ± 14.04^bc^	67.50 ± 3.99^bc^	33.83 ± 2.88^bc^	22.00 ± 3.24^c^	38.33 ± 3.47^bc^
BTE-60	361.50 ± 30.23^ac^	130.67 ± 6.33^ac^	63.50 ± 3.61^ac^	30.50 ± 3.99^c^	64.83 ± 3.24^ac^
RTE-30	5.40 ± 0.68^ab^	2.78 ± 0.26^ab^	0.87 ± 0.18^ab^	1.28 ± 0.08^ab^	1.60 ± 0.18^ab^

^a^p < 0.05 compared to BTE-30; ^b^p < 0.05 compared to BTE-60; ^c^p < 0.05 compared to RTE-30.

## 4 Discussion

Baseline FBG, OG1 & OG2 readings indicate that all the STZ-NA administered rats successfully developed DM (Table 1). After treatment with standard drugs and test drugs, significant reduction in FBG, OG1 and OG2 readings when compared to DC group was observed. Like previous studies [7] the turmeric extracts used in this study also showed significant antidiabetic activity by reducing the blood glucose readings (FBG, OG1 and OG2). MET can show blood glucose lowering activity only after 1–2 weeks of drug administration in humans [18]. In this model, test drugs were administered for 30 days and MET showed antidiabetic activity after 30 days of drug administration (Table 1).

After treatment, FBG, OG1 and OG2 readings were found to significantly higher in RTE-30 treated groups when compared with BTE-30 and BTE-60 treated groups as mentioned in results section. This observation gives the inference that RTE-30 group has lower blood glucose lowering effect/antidiabetic effect when compared with BTE treated groups (Table 1).

Even though the antidiabetic effect of curcumin has been reported in several studies, this is the first study where effect of curcumin on HOMA IR, %S, %B has been demonstrated. The increase in IR in DC group when compared with NC group indicates that the STZ-NA doses used in this study were able to produce type 2 diabetes experimentally in Wistar rats. In this study, bioavailability of curcumin and its derivatives in pancreas after administering RTE and BTE turmeric extracts for 30 days has also been studied in STZ-NA induced diabetic model. When compared with RTE-30 group, BTE treated groups had significantly elevated curcumin levels (Table 4).

There have been several attempts to improve the pharmacokinetics of curcumin. Piperine from black pepper is known to reduce the metabolism of curcumin in the liver by inhibiting liver enzymes. Addition of piperine to turmeric has shown to increase oral absorption of curcumin up to 3 h after which it reaches baseline levels [19]. Curcumin is also complexed with phospholipids to form micelles which can be taken up by cell membranes. But the approach failed to deliver detectable levels of free curcumin in plasma samples after oral intake [20]. Another approach employed in the curcumin pharmacokinetic studies is to use glucuronidase/sulfatase enzyme during the plasma extraction process. It breaks the conjugated curcumin metabolites to liberate curcumin during the extraction process leading to detection of curcumin by HPLC or LCMS methods. These levels are not a true representation of curcumin oral bioavailability as the conjugated metabolites are inactive [21,22]. Addition of emulsifiers and surfactants make turmeric extract well-dispersed in a liquid matrix which is often wrongly interpreted as water solubility. Such formulations use a greater percentage of excipients compared to curcumin in the final formulation leading to higher dose. The real challenge with curcumin oral formulation is to deliver the active form of free curcumin to target organs. The very low levels of curcumin detected in plasma may not be sufficient to provide therapeutic benefits at target organs [23]. The correlation of antidiabetic effects coupled with the improvement of concentration of free curcumin, DMC and BDMC in pancreas explains the higher efficacy of BTE compared to RTE.

HOMA-IR level is a clinical representative of insulin resistance. Turmeric treated groups were found to have decreased IR and increased % B when compared with DC group (Table 2). IR elevation and pancreatic beta-cell dysfunction are main features of type 2 DM. IR is characterized by impaired insulin-mediated glucose utilization in insulin-sensitive tissues and elevated glucose production by liver [24] whereas beta-cell dysfunction appears when ITBackspaceR cannot be compensated by pancreatic beta cells [25].

Turmeric extract treated groups in this study demonstrated weight-lowering effect (Table 3). Curcumin/turmeric supplementation decreases IR by increasing the glucose uptake and oxidation of fatty acids in skeletal muscle and can contribute to the weight-loss [26]. Maximum weight loss was observed in DC group, which correlates with disease activity. MET is known to cause weight loss and it is a known adverse effect of MET. In our study metformin treated rats have developed weight loss during the treatment period. In GLIB treated group, weight gain was observed, it correlates with insulin secretagogue activity of GLIB and anabolic action of released insulin. The effect of MET and GLIB on body weight of rats in this experimental model is mimicking the changes produced by GLIB & MET in humans.

GLIB and MET treated groups showed significant blood glucose lowering effect when compared with DC group. Though the blood glucose readings of GLIB group were found to be high when compared with BTE-60 treated group, this difference was not statistically significant (Table 1). OG1 reading of MET treated group was found to be significantly high (p < 0.05) when compared with BTE-60 group; this observation can be corelated to the low insulin levels found in MET group (Table 1, Table 2).

Turmeric extract treated groups demonstrated antidiabetic effect in this study (Table 1). The antidiabetic effect of turmeric extract can be explained by the presence of free curcumin in pancreas (Table 4). Free curcumin in pancreas can provoke glucose uptake by increasing gene expression of GLUT2 stimulating insulin secretion [27]. Curcumin can also depolarize the plasma membrane of pancreatic beta cells and it can promote insulin release [28,29]. Curcuminoids and essential oils of turmeric have been identified as agonists for peroxisome proliferator–activated receptor-gamma (PPAR-γ) and can lower insulin resistance [30,31]. Hence, these mechanisms might have led to blood glucose lowering effect.

In RTE-30 group serum insulin was found to less than DC group (not significantly significant), but beta-cell functioning and insulin sensitivity was found to be higher than DC group (Table 2). In this group, low insulin levels have not corelated with significant hyperglycemia (Table 1), improved beta-cell functioning in response to elevated blood glucose might have contributed for the blood glucose lowering effect.

## 5 Conclusion

Curcumin and its derivatives present in turmeric reduces blood glucose level by decreasing IR, increasing %S and also by improving %B. Poor bioavailability of curcumin limits the therapeutic effect of RTE. Concentration of curcumin and its derivatives detected in pancreas were significantly higher in BTE treated groups than RTE group. BTE treated groups showed significant antidiabetic or blood glucose lowering effect. Hence, it can be concluded that, this study has shown the increased efficacy and bioavailability of curcumin in BTE over RTE.

## Source(s) of funding

Research was done with Department of Pharmacology fund of Kasturba Medical College, Mangalore.

## Conflict of interest

None.
