# Supplementary figures and images for: Antidiabetic effect of bio-enhanced preparation of turmeric in streptozotocin-nicotinamide induced type 2 diabetic Wistar rats
Source: J Ayurveda Integr Med. 2021 Aug 2;12(3):474–9. doi: 10.1016/j.jaim.2021.04.010 (PMC8377175; doi:10.1016/j.jaim.2021.04.010)

12:01:30

1: MRM of 6 Channels ES+  
309.096 > 90.917 (BISDEMETHOXYCURCUMIN)  
1.71e5

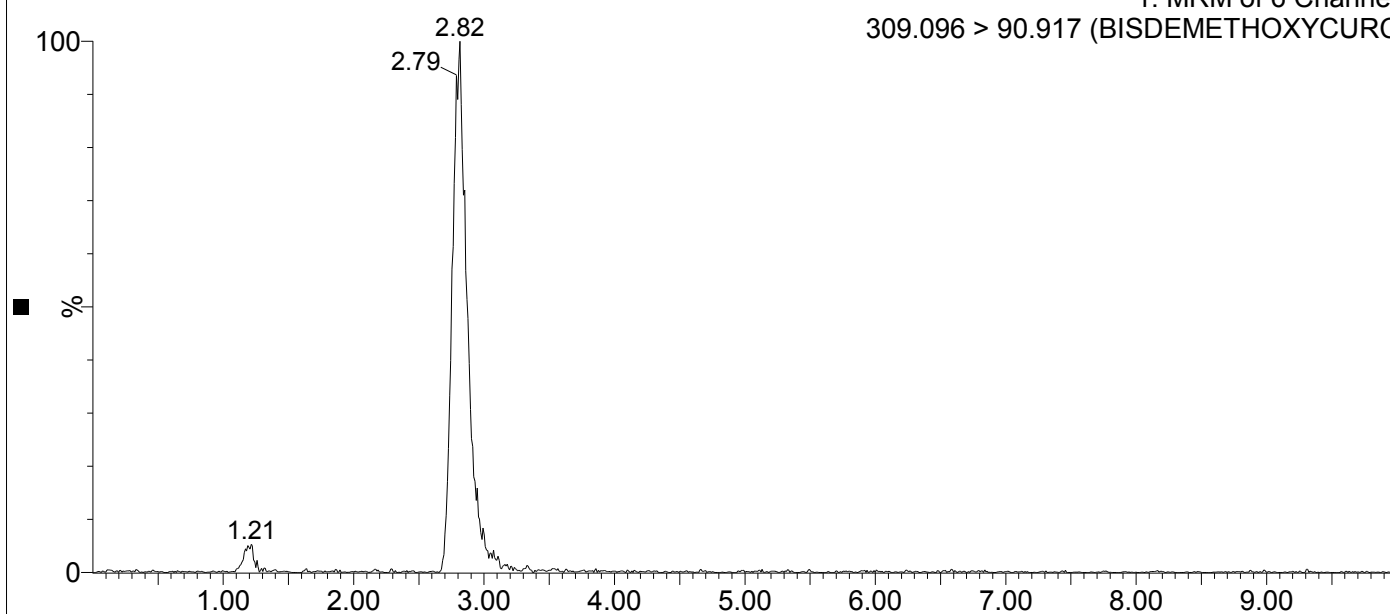

1: MRM of 6 Channels ES+  
339.032 > 146.886 (DMC)  
2.29e5

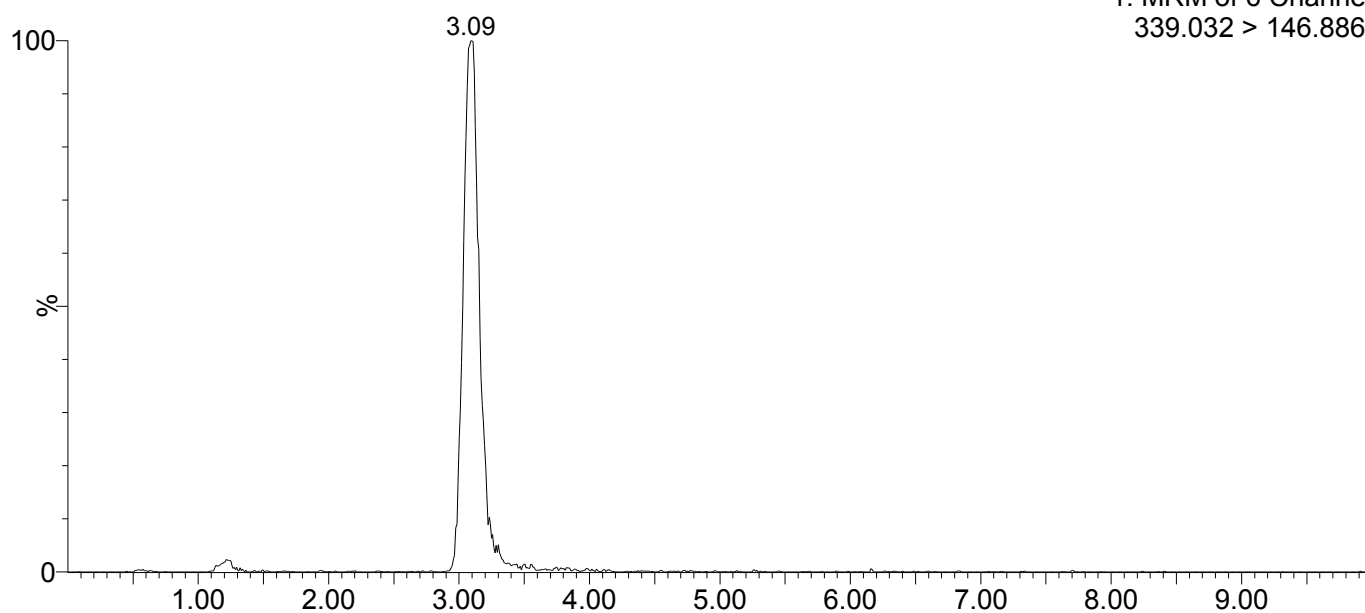

1: MRM of 6 Channels ES+  
369.032 > 176.93 (curcumin)  
6.84e5

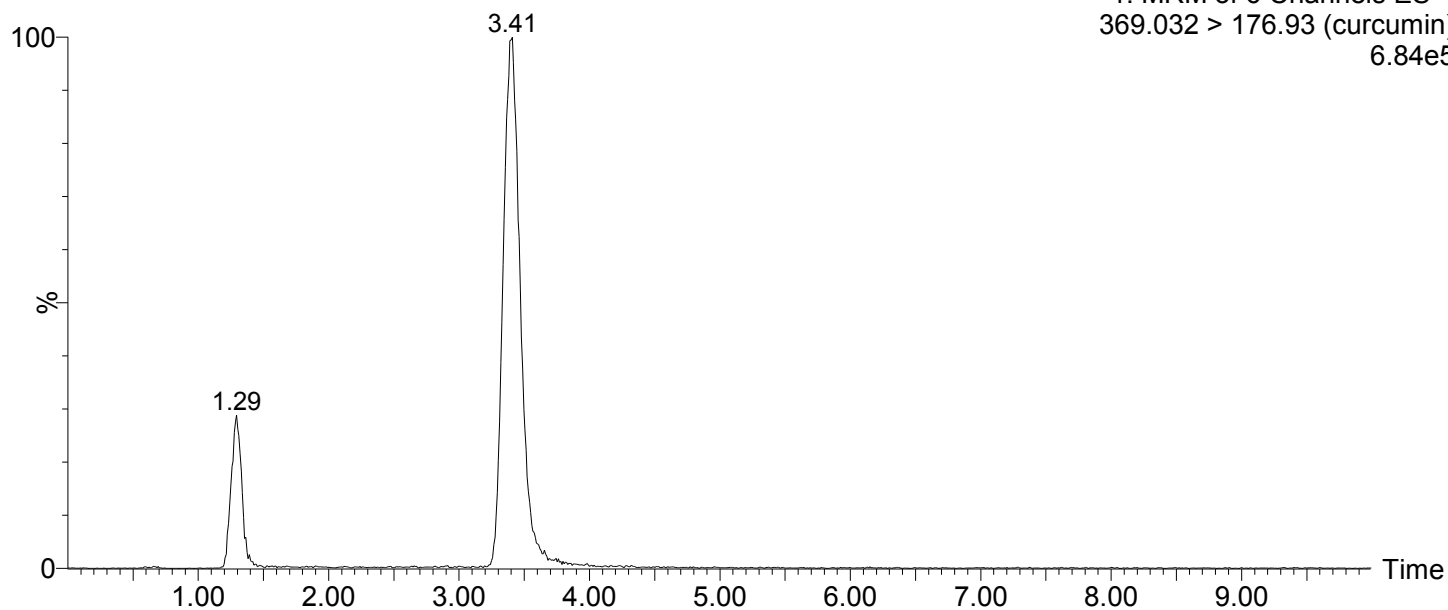

Supplement: Multimedia component 1 [file mmc1.pdf]

14:17:29

2: MRM of 10 Channels ES-  
447.032 > 148.95 (curcumin sulfate)  
6.28e3

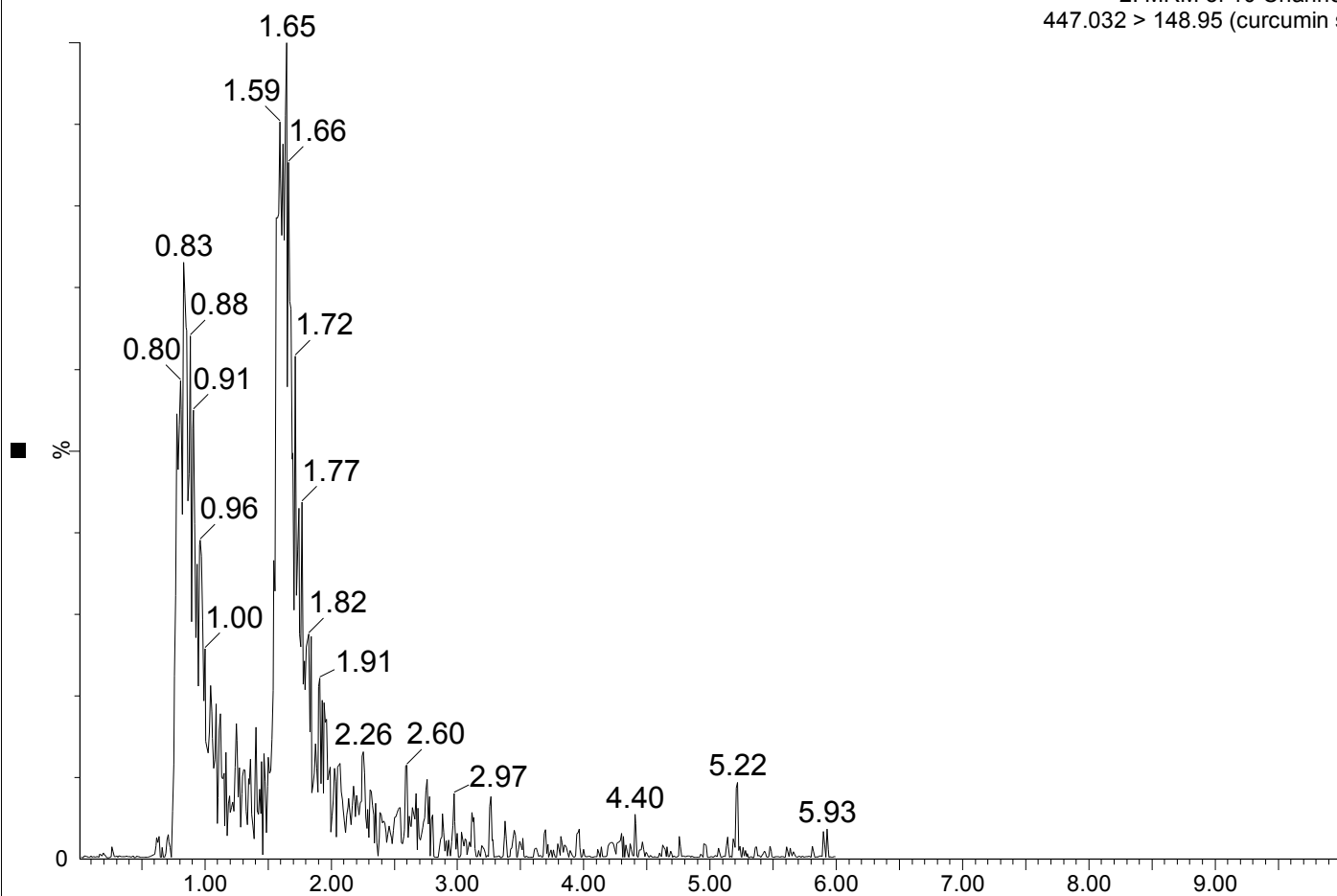

2: MRM of 10 Channels ES-  
543.096 > 148.946 (curcuminglucuronide)  
1.09e5

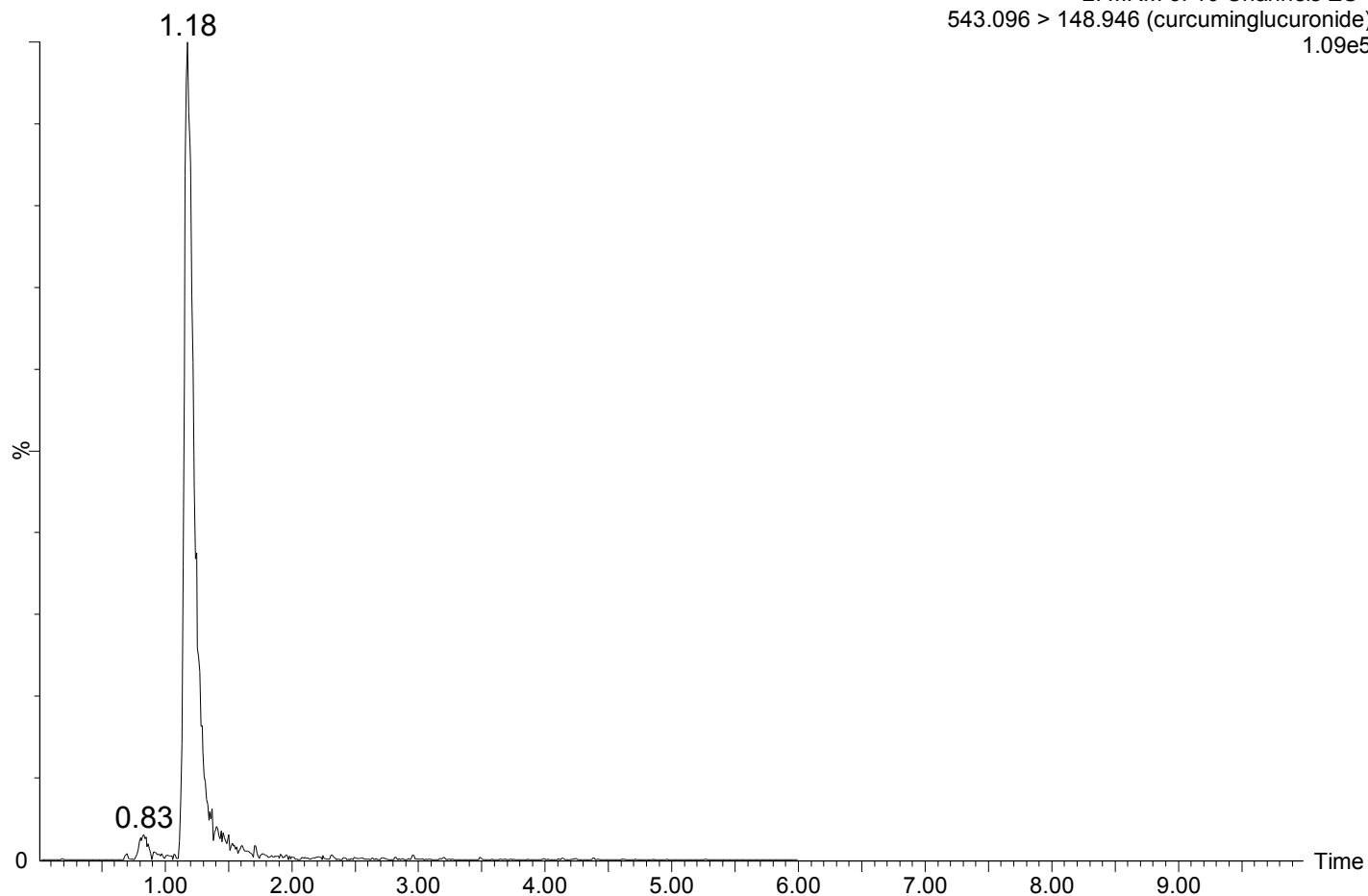

Supplement: Multimedia component 2 [file mmc2.pdf]
